# Single‑center weakly supervised deep learning prediction of KRAS, NRAS, BRAF, and HER2 status in colorectal cancer from histopathology images using internal cross‑validation

**DOI:** 10.1186/s12935-026-04211-8

**Published:** 2026-02-01

**Authors:** Xiang Zhang, Shuangshuang Wang, Qing Gu, Yuchen Fu, Hui Li, Jinwei Gan, Juan Du, Lele Chu, Xiuqing Li, Chenxi Wang, Li Li, Xuya Yuan, Yuan Li, Yi Zhang, Yifen Zhang, Yugen Chen

**Affiliations:** 1 Department of Pathology, Jiangsu Province Hospital of Chinese Medicine, Affiliated Hospital of Nanjing University of Chinese Medicine, Nanjing, China; 2 State Key Laboratory for Novel Software Technology, National Institute of Healthcare Data Science at Nanjing University, Nanjing, China; 3 Comprehensive Cancer Center of Drum Tower Hospital, Medical School of Nanjing University, Clinical Cancer Institute of Nanjing University, Nanjing, China; 4 Department of Colorectal Surgery, Jiangsu Province Hospital of Chinese Medicine, Affiliated Hospital of Nanjing University of Chinese Medicine, Nanjing, China

**Keywords:** Weakly supervised deep learning, Colorectal cancer, Whole slide images, KRAS, NRAS, BRAF, HER2

## Abstract

Research has shown that mutations in the *KRAS*, *NRAS*, and *BRAF* genes are linked to resistance to anti-EGFR therapies in colorectal cancer (CRC) patients. HER2-targeted therapies are increasingly being recommended for individuals with HER2 overexpression. The evaluation of *KRAS*, *NRAS*, *BRAF*, and *HER2* statuses has become an important part of precise diagnosis for CRC. However, conventional molecular or protein testing can be time-consuming and expensive. This study aims to predict the status of *KRAS*, *NRAS*, *BRAF*, and HER2 through the analysis of whole-slide pathology features from CRC samples stained with Hematoxylin-Eosin (H&E) for *KRAS*, *NRAS*, and *BRAF*, and by utilizing Immunohistochemistry (IHC) for HER2. In this study, 435 CRC patients were enrolled from Jiangsu Province Hospital of Chinese Medicine. Using the clustering-constrained attention-based multiple-instance learning (CLAM) model, we constructed four models for predicting the statuses of *KRAS*, *NRAS*, *BRAF*, and HER2 based on whole-slide images (WSIs). This single‑center study used patient‑level internal cross‑validation to train and evaluate weakly supervised CLAM models for predicting KRAS, NRAS, BRAF, and HER2 status from whole‑slide images. The mean area under the receiver operating characteristic (ROC) curve (AUC) values (95% CI) were KRAS 0.8958 (0.8575, 0.9340), NRAS 0.9367 (0.8893, 0.9829), BRAF 0.9876 (0.9744, 1.0000), and HER2 3 + versus non‑3 + 0.99 (0.98–1.00). Given the extremely small NRAS+ (*n* = 14) and BRAF+ (*n* = 21) cohorts, these estimates are statistically fragile and should be interpreted as hypothesis‑generating pending external validation. Our model-generated heatmaps showing *KRAS*, *NRAS*, *BRAF* mutation patterns and HER2 expression levels generally matched the regions identified by the pathologists. This method provides new insights to predict gene mutations and protein expression using deep learning. This single-center study used patient-level internal cross-validation. Robustness and clinical applicability cannot be assumed without external, multi-center validation, and the present results should be interpreted as hypothesis-generating.

## Introduction

Colorectal cancer (CRC) was the third most common cancer and ranked as the second leading cause of cancer-related deaths globally in 2022, as reported by GLOBOCAN [1]. In China, CRC accounts for 30% of both global cases and deaths [2]. About 20% of patients are initially diagnosed with metastatic CRC (mCRC), while up to 50% of patients initially diagnosed with localized disease are likely to progress to metastatic disease [3]. Despite progress in treatment, the prognosis for patients with mCRC remains unfavorable.

Since the early 2000 s, several targeted therapies have been proven effective in treating mCRC and have received FDA approval. Cetuximab and panitumumab are two monoclonal antibodies specifically designed to target the epidermal growth factor receptor (EGFR). These agents have demonstrated efficacy, both as monotherapies and in combination with chemotherapy [4]. Studies have reported that mutations in the *KRAS*, *NRAS* and *BRAF* genes are linked to resistance against anti-EGFR therapies [5]. In CRC patients, approximately 30% to 55% have *KRAS* gene mutations, about 1% to 6% have *NRAS* gene mutations, and 8% to 14% have *BRAF* gene mutations [6]. The National Comprehensive Cancer Network (NCCN) Guidelines also recommend that mCRC patients undergo testing for *KRAS/NRAS/BRAF* mutations before starting anti-EGFR therapies [7]. Approximately 3% of CRC cases has HER2 amplification or overexpression. HER2-targeted therapies are increasingly suggested as treatment options for patients harboring HER2 overexpression [8]. The NCCN Guidelines recommend that all patients with mCRC should be tested for HER2 amplifications [7].

Current methods for molecular or protein testing include next-generation sequencing (NGS), amplification refractory mutation system-polymerase chain reaction (ARMS-PCR) and Immunohistochemistry (IHC). However, their high costs, extended sequencing time, and the varying levels of proficiency among physicians in slide interpretation limit their universal application. Recently, the analysis of pathological slides has increasingly utilized whole slide images (WSIs). Moreover, the development of deep learning (DL)-based artificial technology (AI) has made it possible to perform complex image classification tasks across different medical fields. Numerous studies have highlighted the potential of DL-based methos for predicting genetic level directly from WSIs [9–11]. These methods offer low costs and high efficiency, making them suitable for widespread adoption.

Our study aims to predict the status of *KRAS*, *NRAS*, *BRAF*, and HER2 through the analysis of whole-slide pathology features using a DL model applied to CRC samples stained with H&E for *KRAS*, *NRAS*, and *BRAF*, while employing IHC for HER2. By comparing the predictions by the DL model with assessments from expert pathologists, we aim to evaluate its potential as a cost-efficient solution for real-world clinical applications.

## Methods

### Datasets

**Fig. 1:**
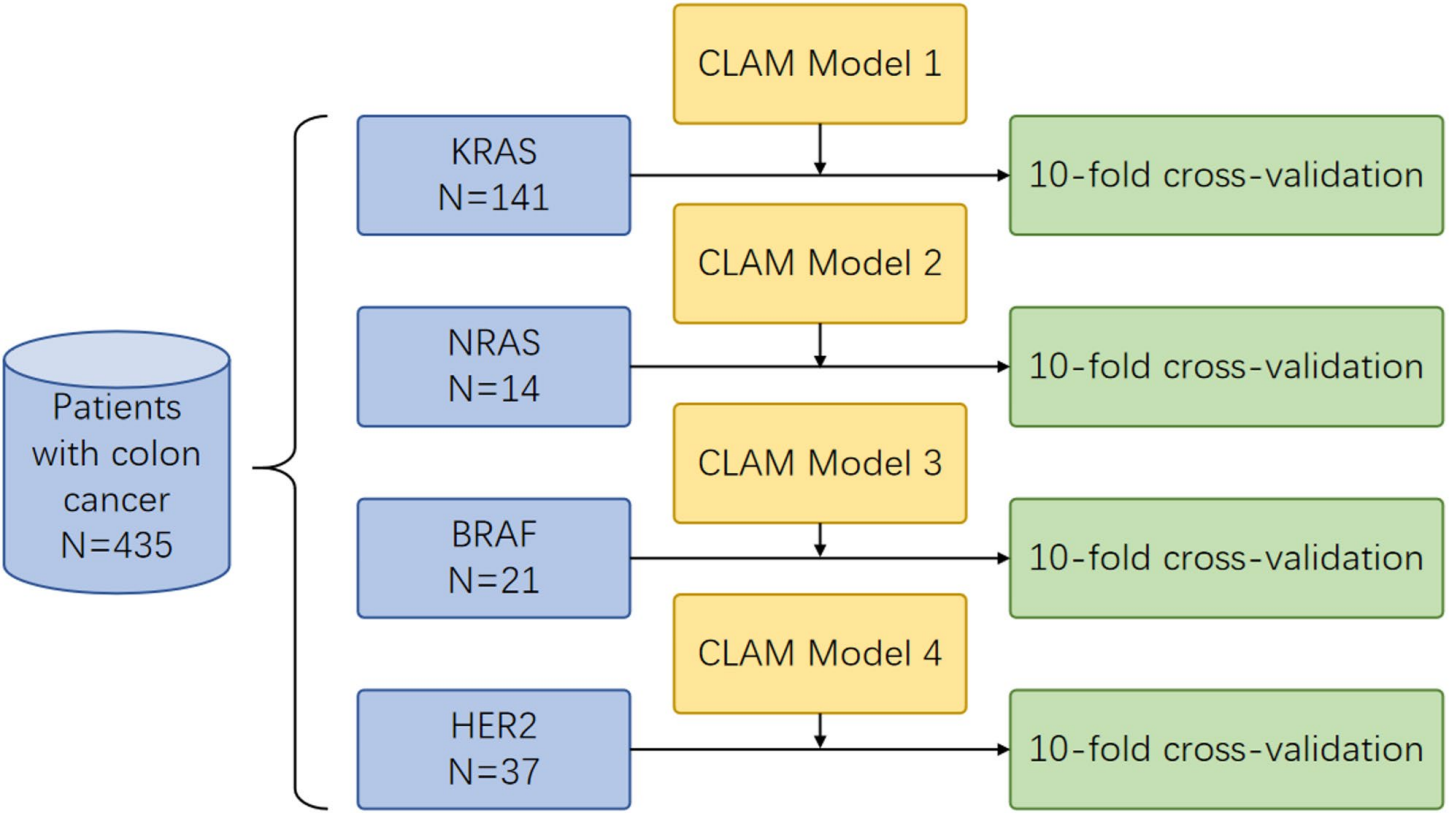
Flow chart of the study. The dataset included 435 patients from Jiangsu Province Hospital of Chinese Medicine, comprising 141 positive cases in the *KRAS* model, 14 in the *NRAS* model, 21 in the *BRAF* model, and 37 cases in HER2 model

With approval from Jiangsu Province Hospital of Chinese Medicine (JSPHCM), Nanjing, China, this study included a total of 435 CRC patients between January 2016 and December 2023. The inclusion criteria included patients with primary CRC. The exclusion criteria included patients who did not have information on KRAS, NRAS, and BRAF gene status, or who lacked continuous tissue sections for H&E-stained WSIs and IHC-stained WSIs for HER2. For the models of KRAS, NRAS, BRAF gene mutations, and HER2 expression, a total of 141, 14, 21, and 37 positive patients were enrolled, respectively (Fig. 1). Reference labels were derived from adjudicated clinical reports using targeted NGS or ARMS-PCR. The most recent adjudicated result per patient served as ground truth. ‘Mutation-positive’ was any pathogenic/likely pathogenic coding variant, and others were negative. Assays followed institutional validation (NGS low single-digit VAF sensitivity and ARMS-PCR manufacturer-validated hotspots).

HER2 IHC staining was performed using a monoclonal rabbit anti-human antibody (Dako A/S, Denmark). HER2 IHC was scored as 0, 1+, 2+, or 3 + by three pathologists. We used the four-class IHC labels (0/1+/2+/3+) and a binary label (3 + vs. non-3+) derived after adjudication with fluorescence in situ hybridization (FISH). In accordance with clinical practice, 2 + cases underwent reflex FISH. A total of 43 IHC 2 + cases underwent FISH testing, and all were determined to be negative. H&E WSIs from primary colorectal cancer resections were included. One representative WSI per patient was analyzed. Only primary tumor tissue was considered and metastatic sites were excluded. Patient-level KRAS, NRAS, BRAF, and HER2 status derived from adjudicated clinical reports served as slide labels without multi-slide aggregation.

## Image annotation

All WSIs were digitized using a 20× objective lens, with a resolution of approximately 0.5 μm per pixel. Annotation was performed using the HistomicsUI (Kitware, Inc., Clifton Park, NY, USA). The annotation content was divided into two parts: (1) annotating the tumor tissue regions in H&E WSIs and (2) annotating the HER2-positive areas in IHC-stained sections. This study received a waiver of consent approved by the Ethics Committee of Jiangsu Province Hospital of Chinese Medicine. Data de-identification procedures were rigorously applied to maintain patient confidentiality. A random sample of WSIs was re-reviewed by a qualified pathologist to ensure the accuracy and integrity of the annotations.

## Data processing

To address the variability caused by different staining methods and imaging devices, we employed Vahadane’s method to normalize the color of all patches [12]. This technique decomposes images into stain density maps through an unsupervised process. By integrating these stain density maps with the stain color basis, the method adjusts the color while maintaining the structural information. While normalization was consistently applied across all images, a quantitative assessment to confirm consistency of the normalization process across train, validation, and test splits was not feasible within the scope of this retrospective analysis. The analysis of WSIs presents distinct challenges because of their large size and high resolution. To address this, each tissue was processed through a patching method using Otsu’s method [13]. This process involves dividing the tissue pixels into smaller patches of size 256 × 256. The number of patches per slide varied with tissue area. Background was excluded during segmentation, and patches with low foreground proportion were discarded. No additional filters for necrosis, mucus, or artifacts were applied.

The feature extraction process utilized a pre-trained image encoder known as UNI, which is based on the Vision Transformer (ViT) architecture [14]. UNI extracts distinctive characteristics from each patch, encoding them into a one-dimensional feature vector of length 1024. We enhanced data diversity by performing operations such as rotation, scaling, and flipping on small patches. Additionally, we standardize the feature vectors to prepare them for processing by DL models.

## Deep learning model

CLAM is a weakly supervised DL approach designed for the efficient analysis and interpretation of WSIs. The CLAM model uses attention mechanisms and patch-level clustering to determine labels at the bag level [15]. CLAM used a 256-dimensional attention layer with dropout 0.25, default clustering with k = 10 centers per class and top-K instance mining, and a composite loss of bag-level cross-entropy and instance-level smooth SVM. Optimization used Adam with a learning rate of 2 × 10⁻⁴, class-balanced sampling, early stopping, and an effective batch size of one slide. The pretrained feature extractor remained frozen, and only attention and classification layers were trained. Input feature dimension was 1024. Features were computed using the UNI-v1 ViT-L checkpoint. Input patches were 256 × 256 pixels. Vahadane stain normalization with a single template was applied identically across training, validation, and test sets. Each patch was encoded into a 1024-dimensional vector for downstream CLAM aggregation. During the training process, the model evaluated each individual patch, assigning scores that reflect the contribution of each patch. To further optimize the feature space and improve data efficiency, CLAM uses instance-level clustering, ensuring that image patches of the same category are grouped together while patches of different categories are separated. It also employs a pseudo-label generation mechanism, where image patches with high attention scores are treated as positive samples, and patches with low attention scores are considered negative samples. CLAM uses a smooth SVM loss function to optimize the model, training it with both patch-level labels and pseudo-labels. These scores are subsequently employed by CLAM to generate heatmaps, which visually show the tissue regions with the most significant influence on the outcomes. In these heatmaps, variations in color correspond to attention scores: areas of high significance are marked in red, while regions of lesser importance are represented in blue. Through these mechanisms, CLAM is able to achieve high attention scores for predicted positive regions and low attention scores for predicted negative regions, thereby distinguishing between different tumor areas. This helps pathologists understand the decision-making process of the model. Therefore, we adopted this model for our study.

**Fig. 2:**
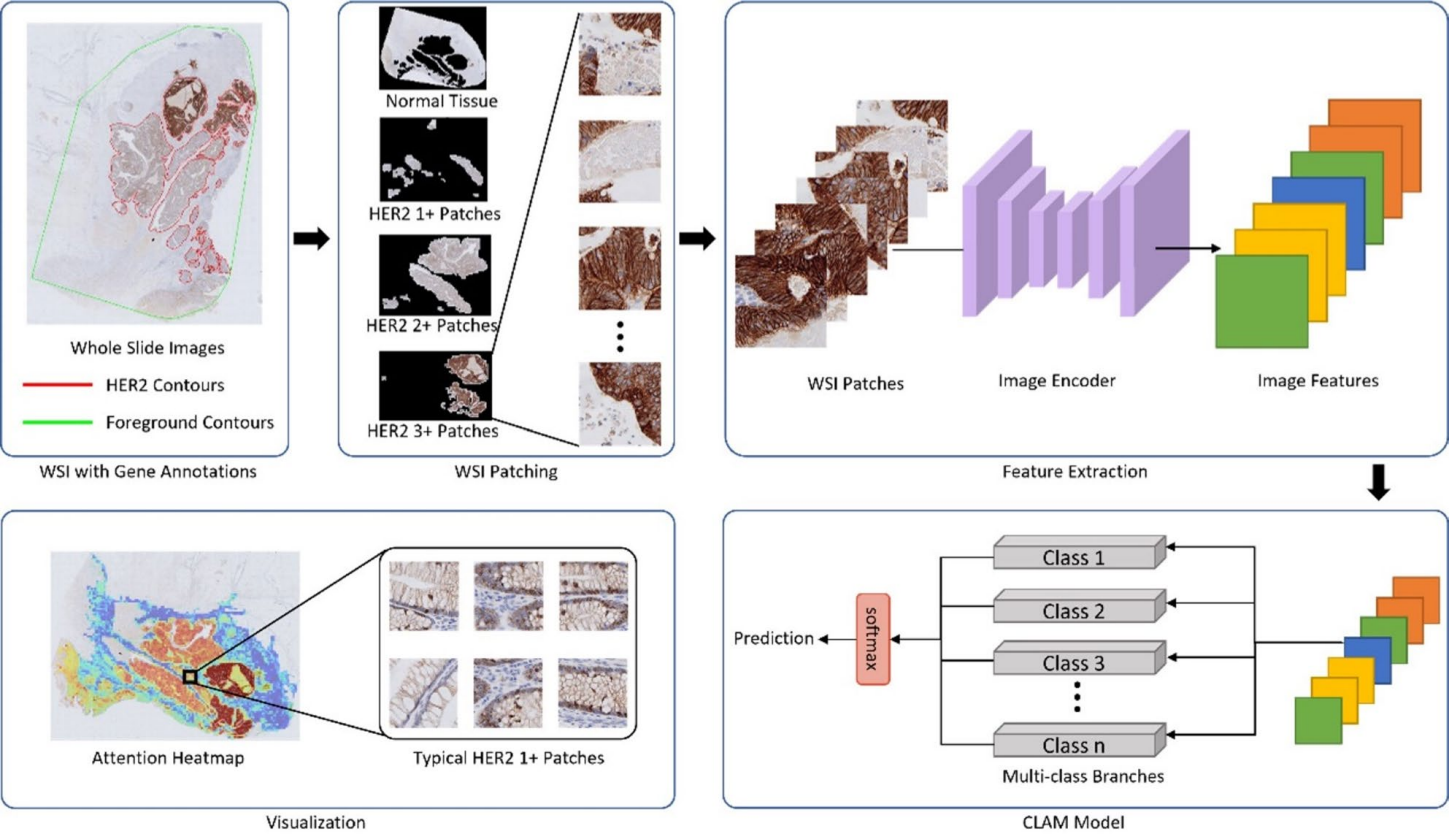
Procedure to develop the clustering-constrained-attention multiple-instance learning (CLAM) model for predicting HER2 expression

For the three genes and HER2 expression, we trained four separate CLAM models. The KRAS, NRAS, and BRAF genes were divided into negative and positive categories, using three binary classification models. HER2 was categorized of 0, 1+, 2+, and 3+, using the multi-class classification model for modeling (Fig. 2).

A sanitized repository is planned with preprocessing code, split scripts with fixed seeds, model training and evaluation pipelines, and configuration files. The repository will operate on de‑identified patch‑level feature files when WSIs cannot be shared. A public repository containing preprocessing, splitting, training and evaluation scripts, fixed random seeds and configuration files will be released to enable reproduction from de‑identified feature files. Whole‑slide images are subject to institutional and privacy restrictions. De‑identified patch‑level features sufficient to reproduce the reported metrics will be made available under a data use agreement.

## Data governance and splitting

All splits were performed at the patient level, ensuring no cross-contamination of WSIs across folds. Each fold utilized an 80%/10%/10% patient stratification that maintained class ratios. A single representative WSI per patient was used for analysis to ensure consistency in model input. HER2 labels were assigned at the patient level after IHC‑to‑FISH adjudication. Splits were performed strictly at the patient level, preventing label leakage and cross‑contamination of WSIs across folds.

### Performance evaluation

A single reporting convention is used throughout. For KRAS, NRAS and BRAF, performance is presented for the mutant versus wild-type classification. For HER2, results are reported for the four-class task with scores 0, 1+, 2+, 3 + and for the binary actionable endpoint of 3 + versus non-3+. We used stratified 10-fold cross-validation performed strictly at the patient level (80%/10%/10% train/validation/test). All WSIs and derived patches from the same patient appeared in only one split within a fold. No patient contributed data to more than one of train, validation, or test in any fold, thereby preventing slide/patch-level leakage. Given class imbalance, we applied label-stratified folds and report the mean area under the receiver operating characteristic (ROC) curve (AUC) as our primary metric, alongside descriptive results for each class. No external validation cohort was available for this study. Furthermore, we compared the attention heatmaps generated by the CLAM model with pathologist annotations of tumor regions to qualitatively assess whether the model highlighted relevant regions.

## Results

### Clinical characteristics of the patients

The clinicopathological characteristics of all 435 CRC patients were analyzed (Table 1). Table 1 showed the clinicopathological characteristics and genetic mutations of all patients (*n* = 435). Among the 298 patients enrolled in KRAS model, 141(47.3%) patients were *KRAS* positive. For the *NRAS* model, 14 (4.7%) were *NRAS* positive. In the *BRAF* model, 21 (7.0%) patients were *BRAF* positive. The HER2 model includes 157 patients, of which 26 (16.6%) has a grade of No staining(0), 51 (32.5%) has a grade of Faint staining (1+), 43 (27.4%) has a grade of Moderate (2+) which in ≥ 50% of cells is tested by FISH, and 37 (23.6%) has a grade of Intense(3+). We report HER2 performance both as a four-class task (0, 1+, 2+, 3+) and as a binary endpoint (3 + vs. non‑3+) following IHC to FISH adjudication, wherein 2 + cases lacking amplification were classified as negative.

**Table 1:** Clinicopathological characteristics and genetic mutations of all patients (*n* = 435)

Characteristic	KRAS(*n* = 298)		NRAS(*n* = 298)		BRAF(*n* = 298)		HER2(*n* = 157)			
	Negative	Positive	Negative	Positive	Negative	Positive	0	1+	2+	3+
Overall, n	157	141	284	14	277	21	26	51	43	37
Age	59.17 ± 11.39	61.48 ± 11.03	60.28 ± 11.38	59.93 ± 9.22	60.68 ± 11.09	54.81 ± 12.29	60.23 ± 12.26	68.04 ± 10.61	64.98 ± 10.65	61.05 ± 9.92
Sex, n (%)										
Male	105	72	170	7	169	8	20	8	27	21
Female	52	69	114	7	108	13	6	23	16	16
Degree of differentiation										
0	4	3	6	1	7	0	2	0	2	2
1	96	93	178	11	178	11	15	33	29	23
2	57	45	100	2	92	10	9	18	12	12
Stage										
1	6	4	10	0	10	0	9	2	7	3
2	15	20	33	2	34	1	4	12	12	8
3	76	64	132	8	132	8	12	33	15	13
4	60	53	109	4	101	12	1	4	9	13

### Predictive efficacy of the proposed deep learning model

CLAM is a weakly supervised approach that leverages attention to identify diagnostically informative regions within whole-slide images (WSIs), thereby enabling accurate slide-level classification. Briefly, CLAM assigns an attention score to each patch to quantify its contribution to the final prediction; these scores are converted to percentiles and scaled from 0 to 1, where higher values indicate greater model focus. Across the 10 folds, class distributions in the test sets were approximately preserved through stratified, patient-level splitting, and analogous stratification was applied to NRAS, BRAF, and HER2 tasks.

**Fig. 3:**
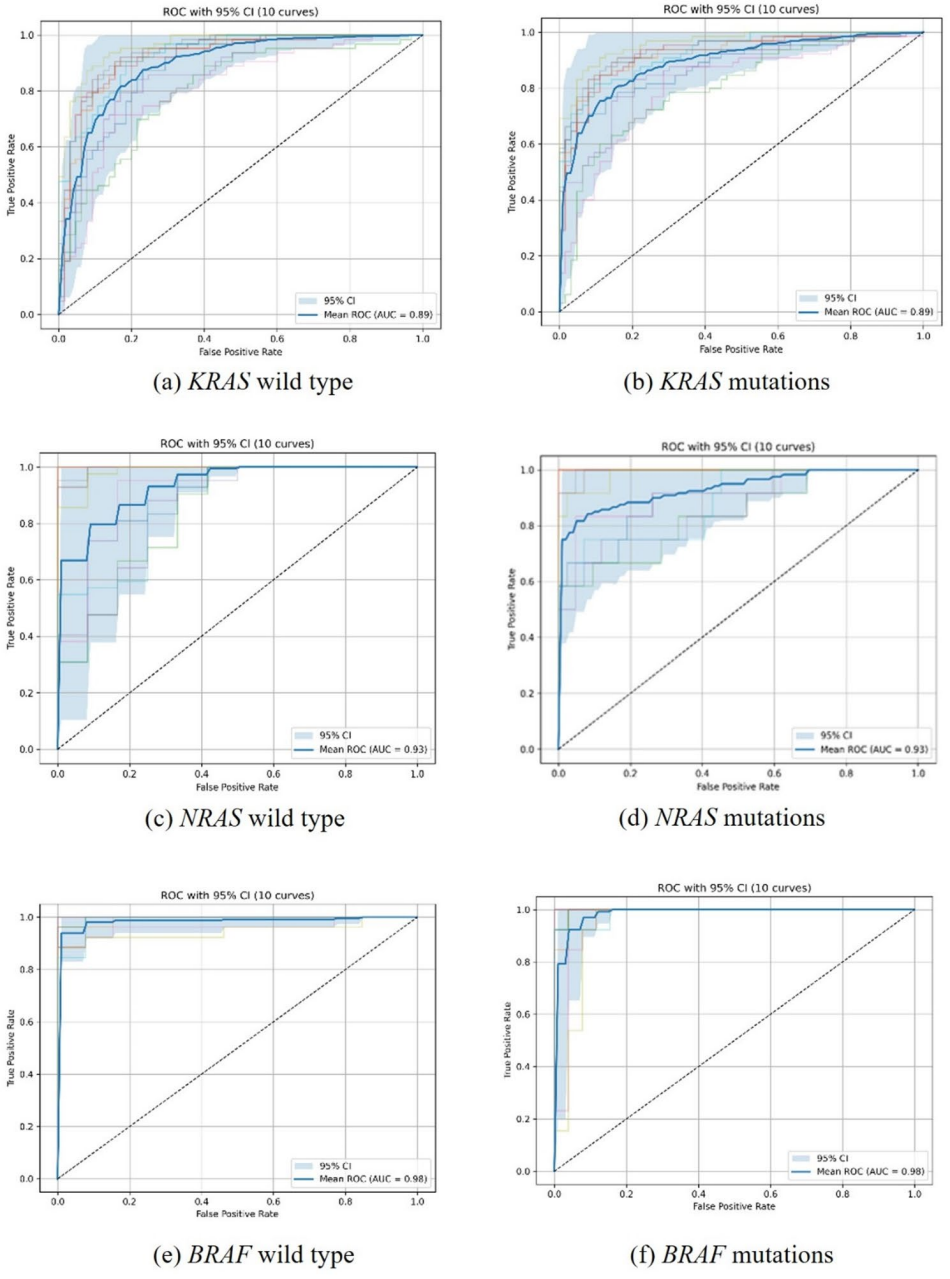
The receiver operating characteristic curve (ROC) of three CLAM models. Model 1 predicted *KRAS* wild type (**a**) and *KRAS* mutations (**b**). Model 2 predicted *NRAS* wild type (**c**) and mutations (**d**). Model 3 predicted *BRAF* wild type (**e**) and mutations (**f**)

To predict KRAS, NRAS, and BRAF status, we trained three binary CLAM models using 435 WSIs from 435 primary CRC cases (298 patients with available labels for each of the KRAS/NRAS/BRAF tasks). Performance was reported consistently as mutant versus wild-type classification, with mean ± SD AUC and corresponding 95% confidence intervals (Fig. 3; Table 2). The KRAS task included 141 mutant and 157 wild-type cases; the NRAS task included 14 mutant and 284 wild-type cases; and the BRAF task included 21 mutant and 277 wild-type cases. Under this unified reporting convention, the mean AUCs (95% CI) were 0.8958 (0.8575, 0.9340) for KRAS, 0.9367 (0.8893, 0.9829) for NRAS, and 0.9876 (0.9744, 1.0000) for BRAF (Fig. 3; Table 2). Given the small numbers of NRAS-positive (*n* = 14) and BRAF-positive (*n* = 21) cases, these AUC estimates may be associated with substantial uncertainty and potential optimism despite patient-level cross-validation; therefore, they should be interpreted as hypothesis-generating and require confirmation in larger, independent cohorts.

**Table 2:** The mean ± SD AUC, 95% CIs, per-class metrics, and test fold counts for each task

Task	AUC(mean ± SD)	AUC 95%CI	Class	test fold counts
KRAS	0.8958 ± 0.0535	[0.8575, 0.9340]	positive	12
	0.8958 ± 0.0535	[0.8575, 0.9340]	negative	12
NRAS	0.9367 ± 0.0654	[0.8893, 0.9829]	positive	5
	0.9367 ± 0.0654	[0.8893, 0.9829]	negative	5
BRAF	0.9876 ± 0.0184	[0.9744, 1.0000]	positive	4
	0.9876 ± 0.0184	[0.9744, 1.0000]	negative	4
HER2	0.9914 ± 0.0027	[0.9896, 0.9982]	normal	10
	0.9911 ± 0.0023	[0.9911, 0.9985]	0	10
	0.9594 ± 0.0122	[0.9432, 0.9811]	1+	10
	0.9798 ± 0.0048	[0.9746, 0.9891]	2+	10
	0.9893 ± 0.0031	[0.9864, 0.9963]	3+	10

**Fig. 4:**
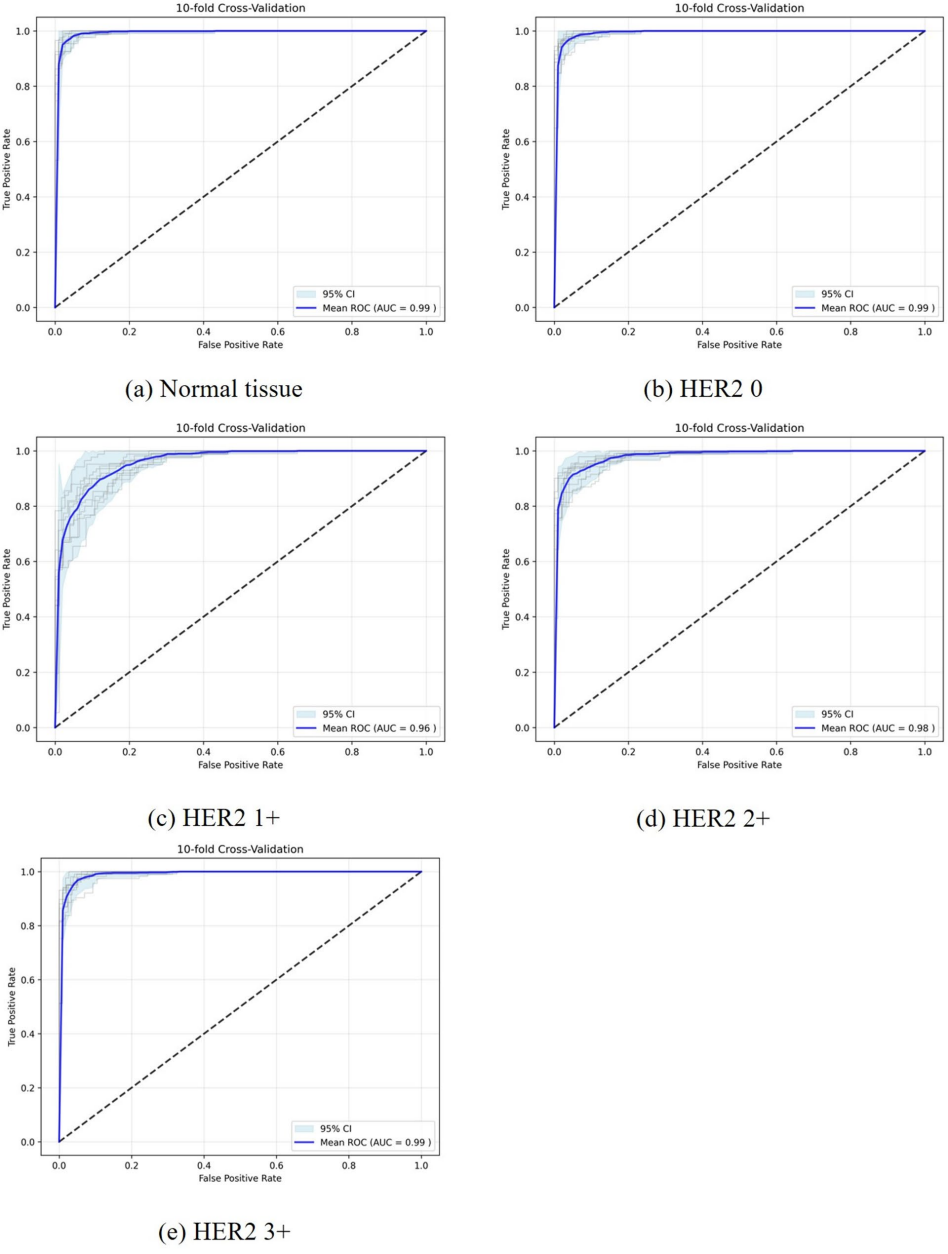
The receiver operating characteristic curve (ROC) of the CLAM model for predicting normal tissue and HER2 scores of 0, 1+, 2+, or 3+

To predict HER2 expression, we developed a multi-class CLAM model using 157 patients, including 26, 51, 43, and 37 cases with IHC scores of 0, 1+, 2+, and 3+, respectively. The model achieved AUCs of 0.9911 (0.9911, 0.9985), 0.9594 (0.9432, 0.9811), 0.9798 (0.9746, 0.9891), and 0.9893 (0.9864, 0.9963) for identifying HER2 scores 0, 1+, 2+, and 3+, respectively, and an AUC of 0.9914 (0.9896, 0.9982) for distinguishing normal tissue and clinically actionable categories (Fig. 4; Table 2).

AUC was selected as the primary evaluation metric because it is threshold-independent and remains informative under class imbalance by reflecting the model’s ranking ability. Consistent with the limited positive sample sizes for NRAS and BRAF, our findings warrant cautious interpretation and should be validated in external datasets.

### Comparative analysis of the CLAM model versus Pathologist-Evaluated assessments

**Fig. 5:**
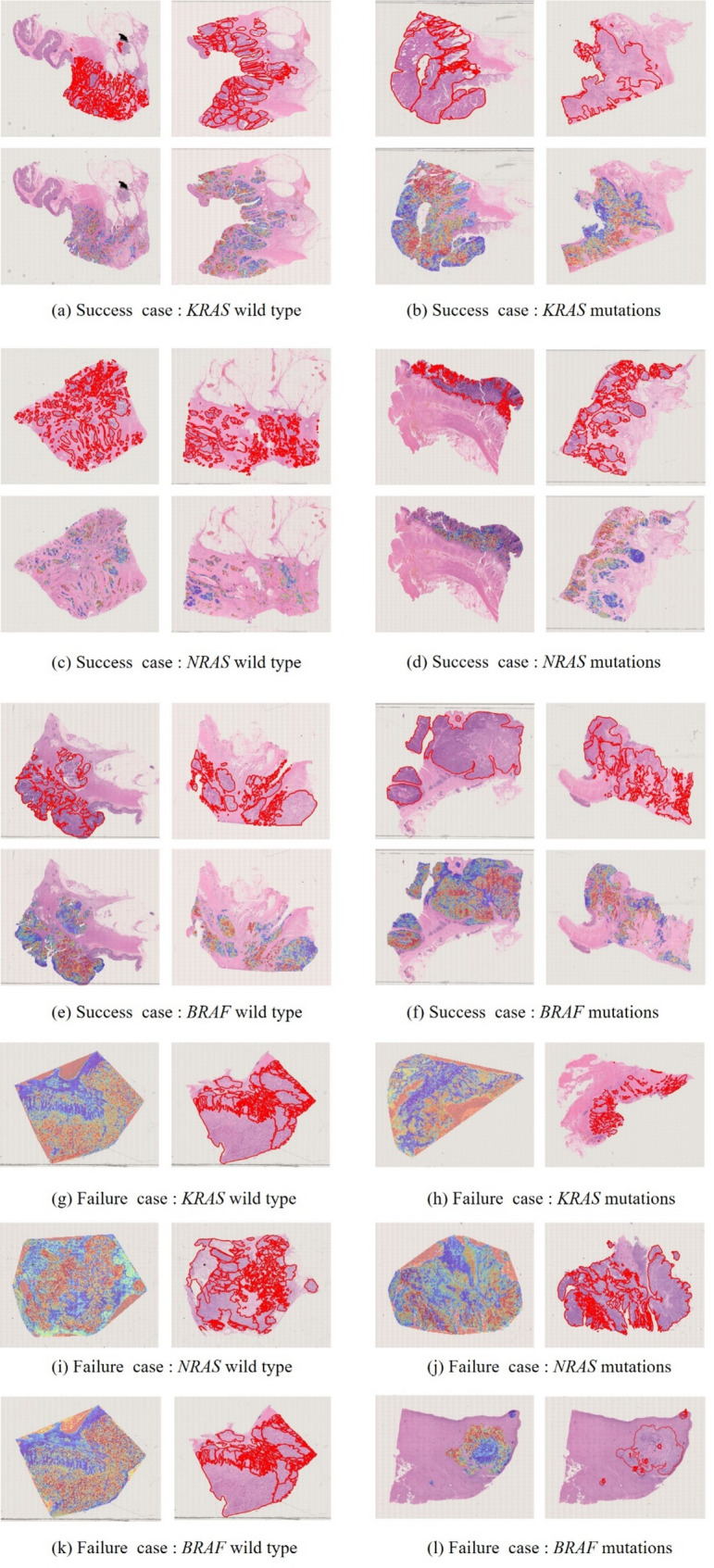
Visualization of patient examples for successfully predicting *KRAS* (**a**, **b**), *NRAS* (**c**, **d**) *and BRAF* (**e**, **f**) mutations, and failure case of per task (**g**-**l**). Each example shows the H&E slides marked by the pathologist with a red line indicating the tumor area in the first row, and the second row displays the heatmap generated by the model, where the red areas represent a larger weight

Based on the performance of the CLAM models described above, we aimed to evaluate their efficacy in comparison to pathologists in predicting mutations in KRAS, NRAS and BRAF genes, as well as HER2 IHC results using HE stained slides. The CLAM model generated heatmaps by identifying and combining areas of WSIs that have high diagnostic significance (indicated by high attention scores represented in red) while disregarding areas with low diagnostic importance (indicated by low attention scores represented in blue) related to the prediction. The distribution of mutations in the BRAF, KRAS, and NRAS genes as shown in the heatmap was generally consistent with the areas marked by the pathologists (Fig. 5).

**Fig. 6:**
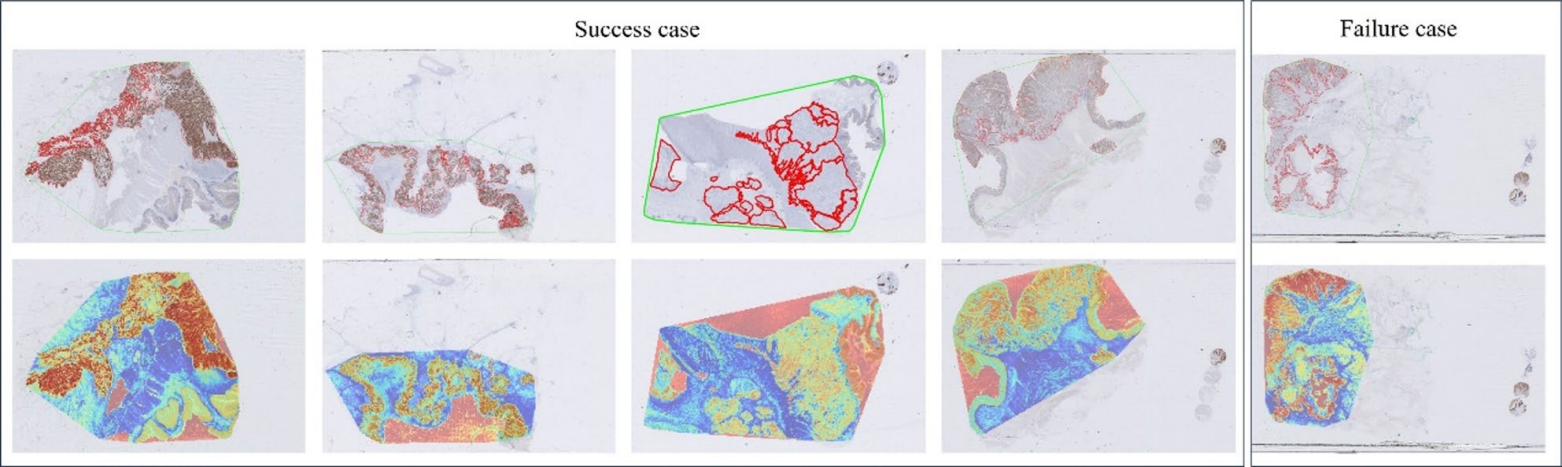
Visualization of patient examples for predicting HER2 expression. Each example displays the IHC slides marked by the pathologist, with a red line indicating the HER2 positive area in the first row, and the second row presents the heatmap generated by the model based on the H&E slides

The heatmap generated by the HER2 prediction model clearly distinguished each region with different colors. Notably, the distribution of HER2 reflected in the attention heatmap aligns closely with the areas marked by pathologists. Furthermore, we observed that the non-tissue regions within the green outlines annotated by the pathologists appear red in the heatmap, demonstrating the model’s robustness in identifying contours (Fig. 6). Attention heatmaps serve as qualitative visualization aids and are not presented as biological explanations. No quantitative spatial agreement analysis or blinded pathologist assessment was conducted in this study.

## Discussion

CRC is the second leading cause of cancer-related deaths worldwide [1]. Testing for KRAS, NRAS, BRAF, and HER2 has emerged as a critical step in the precise diagnosis of CRC [16]. KRAS/NRAS are predictive biomarkers for anti-EGFR therapy. Studies suggest that patients with RAS-mutated CRC who receive cetuximab or panitumumab have significantly shorter survival compared to those with wild-type tumors [17].

BRAF mutations are considered a negative predictive marker for the effectiveness of anti-EGFR therapies. However, they may respond well to a combination of BRAF inhibitors and anti-EGFR antibodies [18]. Additionally, CRCs driven by HER2 can be treated with various HER2 kinase inhibitors [19]. NGS, ARMS-PCR and IHC are undoubtedly the preferred methods for molecular and protein testing, respectively. However, their application is still limited due to high costs and extended turnaround times.

Several studies have examined the application of AI in molecular testing, highlighting the potential to improve cost-effectiveness and efficiency. These investigations have used histopathology images to predict gene status, suggesting a correlation between histopathological morphology and the molecular profile. Li et al. recently developed a model using a convolutional neural network (CNN) to predict the status of KRAS, NRAS, and BRAF genes in patients with left-sided mCRC using H&E-stained histopathological images. The model achieved an AUC of 0.83 in the testing cohort [20]. Yang et al. applied a DNN to predict the HER2 status in breast cancer patients, combining H&E-stained images and clinical data, achieving an AUC value of 0.76 [21].

To our knowledge, this study is among the first attempts to predict the gene mutations (KRAS, NRAS and BRAF) and HER2 expression in CRC using an independent JSPHCM cohort to develop the models. CLAM, a method of weakly supervised learning, has been reported to predict gene status based on histopathological images [22]. Adachi et al. developed the CLAM model to predict p16 expression in oropharyngeal cancer by analyzing histopathological images, achieving an AUC of 0.905 [22]. In this study, we developed four models based on CLAM that can predict the status of KRAS, NRAS, BRAF, and HER2 through the analysis of whole-slide pathology features from CRC samples stained with H&E for KRAS, NRAS, and BRAF, and by utilizing IHC for HER2.

The AUC for predicting mutations in KRAS, NRAS and BRAF genes, as well as HER2 expression, was over 0.88. The models demonstrated strong discrimination, supporting the potential clinical utility of DL-based prescreening. In addition, our data were sourced from the WSIs database of our hospital for CRC, which better aligns with the pathological characteristics of Chinese patients and holds practical application value.

However, this study had several limitations. Exclusive reliance on single-center internal validation limits generalizability. Robustness across institutions, acquisition protocols, and patient populations cannot be inferred without external, multicenter validation. The current findings are institution-specific and hypothesis-generating and require confirmation in independent cohorts. Differences in scanner hardware/software, fixation and processing, section thickness, and region-of-interest selection can shift the histomorphometric feature distribution and degrade performance. The present results should be considered institution-specific and hypothesis-generating. Prospective validation in independent multicenter cohorts with heterogeneous scanners, pre-analytic workflows, and diverse patient populations is planned to establish robustness and transportability. Small positive classes (especially NRAS and BRAF) may increase uncertainty in estimates despite cross-validation. Although we enforced patient-level splits to avoid leakage, we did not perform prospective testing. We did not quantify spatial agreement (e.g., Dice/IoU against tumor masks or HER2-positive ROIs), nor did we obtain blinded Likert-scale ratings of heatmap utility. Moreover, occasional non-tissue false positives may occur despite masking. The four separate models may also complicate deployment. These models are intended as prescreening tools to prioritize confirmatory NGS/IHC rather than to replace standard testing. Future work will focus on multicenter external validation, prospective evaluation, and simplifying to a unified model for clinical use. Interpretability is also limited. Attention maps reflect model saliency and should not be construed as mechanistic or biological explanations. Quantitative spatial agreement and blinded multi-reader assessment are required to substantiate clinical interpretability. Clinical utility remains unproven. Decision thresholds and downstream impact were not formally assessed in this retrospective study, and any implication of workflow integration should be considered provisional pending prospective decision-analytic evaluation.

## Conclusion

In conclusion, we presented four AI models based on H&E-stained histopathological images to predict the status of the KRAS, NRAS, and BRAF genes, as well as HER2 expression assessed through IHC staining. In the analysis framework using CLAM, features were presented in a format that pathologists could easily identify through visualization. This study provides new insights into the clinical application of DL for predicting gene mutations and protein expression. The development of predictive models to evaluate KRAS, NRAS, BRAF, and HER2 status in colorectal cancer presents a promising advancement in precision medicine. Additionally, due to the inconsistencies in the expertise among physicians interpreting H&E and IHC results, we have trained the AI to emulate the assessments of expert pathologists, ensuring consistency in slide interpretation quality. Our intended use is prescreening. However, we did not conduct formal decision-analytic evaluation in this retrospective study. Within these limitations, the results should be interpreted as hypothesis-generating and supportive of prescreening, pending external multicenter validation and prospective studies.

## Data Availability

The data that support the findings of this study are available from the corresponding author upon reasonable request.
